# *Bordetella pertussis* whole cell immunization protects against *Pseudomonas aeruginosa* infections

**DOI:** 10.1038/s41541-022-00562-1

**Published:** 2022-11-10

**Authors:** Catherine B. Blackwood, Margalida Mateu-Borrás, Emel Sen-Kilic, Gage M. Pyles, Sarah Jo Miller, Kelly L. Weaver, William T. Witt, Annalisa B. Huckaby, Jason Kang, Courtney E. Chandler, Robert K. Ernst, F. Heath Damron, Mariette Barbier

**Affiliations:** 1 West Virginia University Vaccine Development Center, Department of Microbiology, Immunology and Cell Biology, 64 Medical Center Drive, Morgantown, WV 26505 USA; 2 University of Maryland, Baltimore Department of Microbial Pathogenesis, School of Dentistry, 650 W. Baltimore St., Baltimore, MD 21201 USA

**Keywords:** Preclinical research, Vaccines, Infectious diseases

## Abstract

Whole cell vaccines are complex mixtures of antigens, immunogens, and sometimes adjuvants that can trigger potent and protective immune responses. In some instances, such as whole cell *Bordetella pertussis* vaccination, the immune response to vaccination extends beyond the pathogen the vaccine was intended for and contributes to protection against other clinically significant pathogens. In this study, we describe how *B. pertussis* whole cell vaccination protects mice against acute pneumonia caused by *Pseudomonas aeruginosa*. Using ELISA and western blot, we identified that *B. pertussis* whole cell vaccination induces production of antibodies that bind to lab-adapted and clinical strains of *P. aeruginosa*, regardless of immunization route or adjuvant used. The cross-reactive antigens were identified using immunoprecipitation, mass spectrometry, and subsequent immunoblotting. We determined that *B. pertussis* GroEL and OmpA present in the *B. pertussis* whole cell vaccine led to production of antibodies against *P. aeruginosa* GroEL and OprF, respectively. Finally, we showed that recombinant *B. pertussis* OmpA was sufficient to induce protection against *P. aeruginosa* acute murine pneumonia. This study highlights the potential for use of *B. pertussis* OmpA as a vaccine antigen for prevention of *P. aeruginosa* infection, and the potential of broadly protective antigens for vaccine development.

## Introduction

Antibiotic-resistant bacterial infections represent a significant burden globally, accounting for more than 2.8 million infections and 35,000 deaths yearly in the U.S. alone^1^. As a result, the threat of antibiotic-resistant infections continues to drive the development of preventative measures such as vaccines^1–3^. Whole cell vaccines train the immune system to mount a response against a specific pathogen, but can also have non-specific immunological effects^4–9^. These non-specific effects can be mediated by both innate and adaptive immune responses to vaccination^9^. In some instances, vaccination protects against other pathogens through the production of cross-reactive antibodies that bind closely-related antigens in other organisms^5,10,11^. For example, anti-Influenza antibodies can protect against heterologous strains of the virus, and exposure to flaviviruses prevents subsequent infection by other flaviviruses^12,13^. Similarly, the fungal *Candida albicans* Hyr1 protein induces a protective response against *Acinetobacter baumannii* surface proteins, via either active and passive immunization^14^. Other examples include Herpesvirus antigens that induce protection against the bacterial pathogens *Listeria monocytogenes* and *Yersinia pestis*^15^.

In addition to protection mediated by cross-reactive antibodies, it is now well-established that whole cell vaccines are able to induce trained innate immunity that leads to epigenetic reprogramming of innate immune cells such as monocytes and macrophages, allowing for a quicker response to exposure to a different pathogen^16–18^. For example, the Bacille Calmette-Guérin (BCG) vaccine for *Mycobacterium tuberculosis*, used in countries where tuberculosis is still prevalent, modulates the innate immune response and induces protection against non-mycobacterial species and some types of cancers^16–19^. Similar effects have been observed with the measles, mumps, and rubella (MMR) vaccine^20,21^.

A growing body of evidence supports that whole cell pertussis vaccines (denoted here as *Bp-*WCV) also provide non-specific protection against other pathogens or act as adjuvant^5–7,9,22,23^. For example, it was recently shown that co-administration of the commercially available Diphtheria, Tetanus, and Pertussis (DTP) vaccine with BCG is associated with increased efficacy of the BCG vaccine and improves overall outcomes for children^9,22,23^. More recently, *Bp-*WCVs have been used to adjuvant vaccines^5–7^. Furthermore, the live-attenuated *B. pertussis* vaccine BPZE1, currently in clinical development phase, is also known to induce a short-term cross-protective response against *Streptococcus pneumoniae*^24^.

Altogether, these data led us to investigate additional and potentially beneficial non-specific effects of the *Bp-*WCV against antibiotic-resistant bacterial pathogens, including the ESKAPE pathogens *Escherichia coli*, *Staphylococcus aureus, Klebsiella pneumoniae, Pseudomonas aeruginosa, A. baumannii*, and *Enterobacter cloacae*. We observed cross reactivity with antibodies induced by *Bp-*WCV but initially focused our efforts on characterizing the response to *P. aeruginosa*. This pathogen is a major causative agent of antibiotic-resistant infections and has no vaccine approved for human use. *P. aeruginosa* causes a broad range of acute and chronic infections that are difficult to treat and are associated with high morbidity and mortality in at-risk patients such as those with cystic fibrosis (CF). In the present study, we determined that *Bp-*WCV vaccination is protective against *P. aeruginosa* in various strains of mice using multiple routes of administration and adjuvant formulations. We identified the *P. aeruginosa* antigens recognized by *Bp*-WCV sera as GroEL and OprF (homologous to GroEL and OmpA in *B. pertussis*), and confirmed that vaccination with *B. pertussis* OmpA protects against *P. aeruginosa* infection. Overall, this work sheds light on how protection mediated by vaccination against one pathogen can be used against another, and identified antigens that can potentially be used for vaccination and protection against multiple bacterial species.

## Results

### Pertussis whole cell immunization induces production of cross-reactive serum antibodies against various bacterial pathogens

**Fig. 1:**
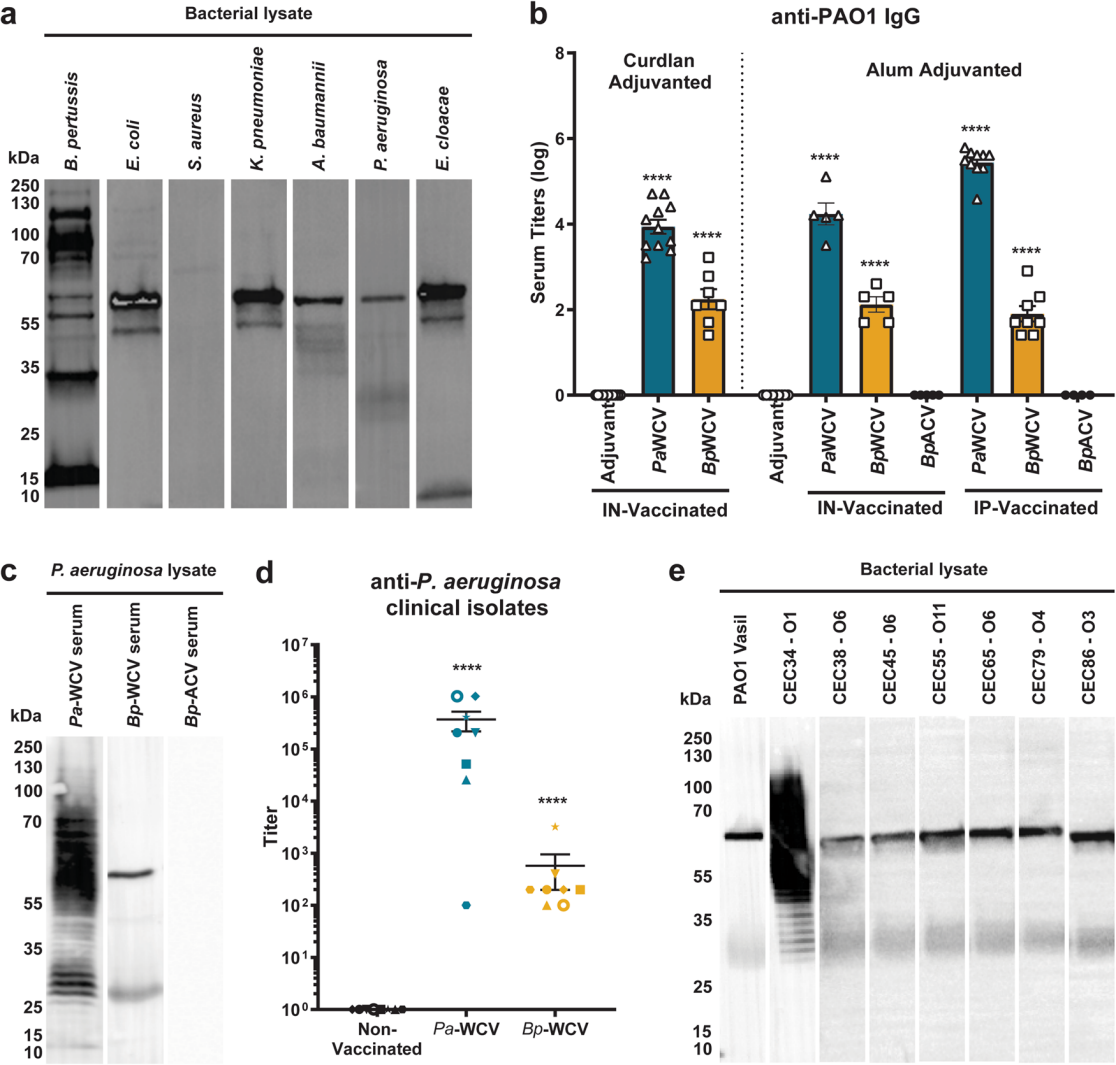
*B. pertussis* whole cell but not acellular immunization induces production of cross-reactive antibodies. **a** Western blot using *Bp-*WCV immunized sera against 10 μg of whole cell *B. pertussis*, *E. coli, S. aureus, K. pneumoniae, A. baumannii, P. aeruginosa,* and *E. cloacae* bacterial lysates. All blots in this panel were performed in parallel. **b** Serum titers determined by ELISA against whole cell *P. aeruginosa* in mice immunized with adjuvant only, *Pa*-WCV, *Bp*-WCV, or *Bp*-ACV, either intranasally (IN) or intraperitoneally (IP). Each dot represents the average titer for a single mouse (*n* = 5–11 mice/group), and error bars represent standard error of the mean. **c** Western blot against *P. aeruginosa* bacterial lysate using *Pa*-WCV, *Bp*-WCV, or *Bp*-ACV sera from pooled IN and IP immunized mice. **d** Serum titers determined by ELISA against clinical isolates of *P. aeruginosa* in mice immunized with adjuvant only, *Pa*-WCV, or *Bp*-WCV pooled serum samples. Each unique dot shape represents average titer against a clinical isolate, and error bars represent standard error of the mean. **e** Western blot against *P. aeruginosa* clinical isolate bacterial lysate using pooled *Bp*-WCV immunized sera. All blots in this panel were performed in parallel. In **b** and **d**, asterisks represent statistical significance, determined by one-way ANOVA. Asterisks over bars indicate comparison to the adjuvant vaccinated. ****p < 0.0001.

Pertussis immunization, in particular whole cell immunization, can trigger off-target or non-specific immune effects^8,9,25^. While the beneficial effects of *Bp-*WCV for the prevention of pertussis are well-established, the protection *Bp-*WCV provides against other pathogens is less understood. Here, we hypothesized that *Bp*-WCV vaccination leads to the production of antibodies that can cross-react with other bacterial pathogens. To test our hypothesis, we immunized 6-week-old female CD-1 mice with the NIBSC standard *B. pertussis* whole cell vaccine adjuvanted with alum and administered by intraperitoneal injection. Mice were boosted at day 21, and serum was collected and pooled on day 35. The pooled *Bp-*WCV serum sample was then tested for cross reactivity towards highly antibiotic-resistant ESKAPE bacteria (*S. aureus, K. pneumoniae, A. baumannii, P. aeruginosa*, and *E. cloacae*), and *E. coli*^26–28^. Using western blotting, we identified that *Bp-*WCV induced antibodies able to bind to at least one antigen in all the Gram-negative species tested (Fig. 1a, Supplemental Fig. 3). However, limited reactivity was detected against the Gram-positive *S. aureus* strain tested (Fig. 1a).

Our group has a long-standing interest in the development of vaccines against *P. aeruginosa*. Despite extensive efforts by the research community including human clinical trials, there is still no vaccine approved for human use. Our laboratory recently showed that B cell and antibody responses against *P. aeruginosa* immunization are essential for protection against acute *P. aeruginosa* pneumonia challenge^29,30^. Therefore, we aimed to determine if the cross-reactive antibody response to *Bp-*WCV immunization could provide new answers for the formulation of a subunit *P. aeruginosa* vaccine.

To determine if vaccination with *Bp*-WCV could provide protection against *P. aeruginosa*, we used outbred CD-1 mice as they are classically used by the pharmaceutical industry to determine vaccine lot efficacy and likely reflect a greater population diversity. CD-1 mice were immunized with *Bp*-WCV and *Pa-*WCV (positive control for protection). We also immunized mice with the acellular pertussis vaccine DTaP Infanrix (*Bp*-ACV) to determine if the production of a cross-reactive immune response to *P. aeruginosa* is specific to *Bp-*WCV or is also provided by other pertussis vaccines. The formulations were adjuvanted with either alum or curdlan, and administered either intranasally or intraperitoneally. We first performed a serological analysis of the immune response to vaccination using ELISA and observed that vaccination with adjuvants alone did not induce the production of anti-*P. aeruginosa* PAO1 IgG. However, both *Pa-*WCV and *Bp-*WCV immunizations induced the production of significant levels of anti-PAO1 serum IgG compared to adjuvant controls (Fig. 1b). No anti-*P. aeruginosa* IgG antibodies were detected in response to DTaP vaccination. Using Western blotting, we observed that *Pa-*WCV vaccination triggered the production of antibodies towards numerous PAO1 antigens, while antibodies present in the *Bp-*WCV sera bound two distinct PAO1 products (Fig. 1c, Supplemental Fig. 4). Again, no binding was detected in the *Bp-*ACV group (Fig. 1c). Taken together, these data indicate that whole cell but not acellular pertussis immunization induces the production of anti-*P. aeruginosa* antibodies.

*P. aeruginosa* infections can be caused by genetically and phenotypically diverse strains. The presence of 20 different known *P. aeruginosa* serotypes and the expression of antigenically variable surface proteins by clinically relevant strains represent a challenge for vaccine development. To evaluate if the antibodies raised in response to *Bp-*WCV vaccination react against non-lab-adapted *P. aeruginosa* strains, we selected a panel of clinical *P. aeruginosa* isolates from patients with cystic fibrosis. The isolates tested include strains expressing LPS from 4 different serotypes, mucoid and non-mucoid isolates, as well as motile and non-motile strains (Supplementary Table 1). We observed that vaccination with *Bp-*WCV led to the production of serum IgG that react with all clinical isolates tested by both ELISA (Fig. 1d) and Western blotting (Fig. 1e, Supplemental Fig. 5), which suggests that the cross-reactive response to *Bp-*WCV against *P. aeruginosa* could be broadly relevant against clinical isolates and serotype-independent.

### *B. pertussis* whole cell immunization induces protection in outbred and inbred mice, and in the β-ENaC murine model for cystic fibrosis

**Fig. 2:**
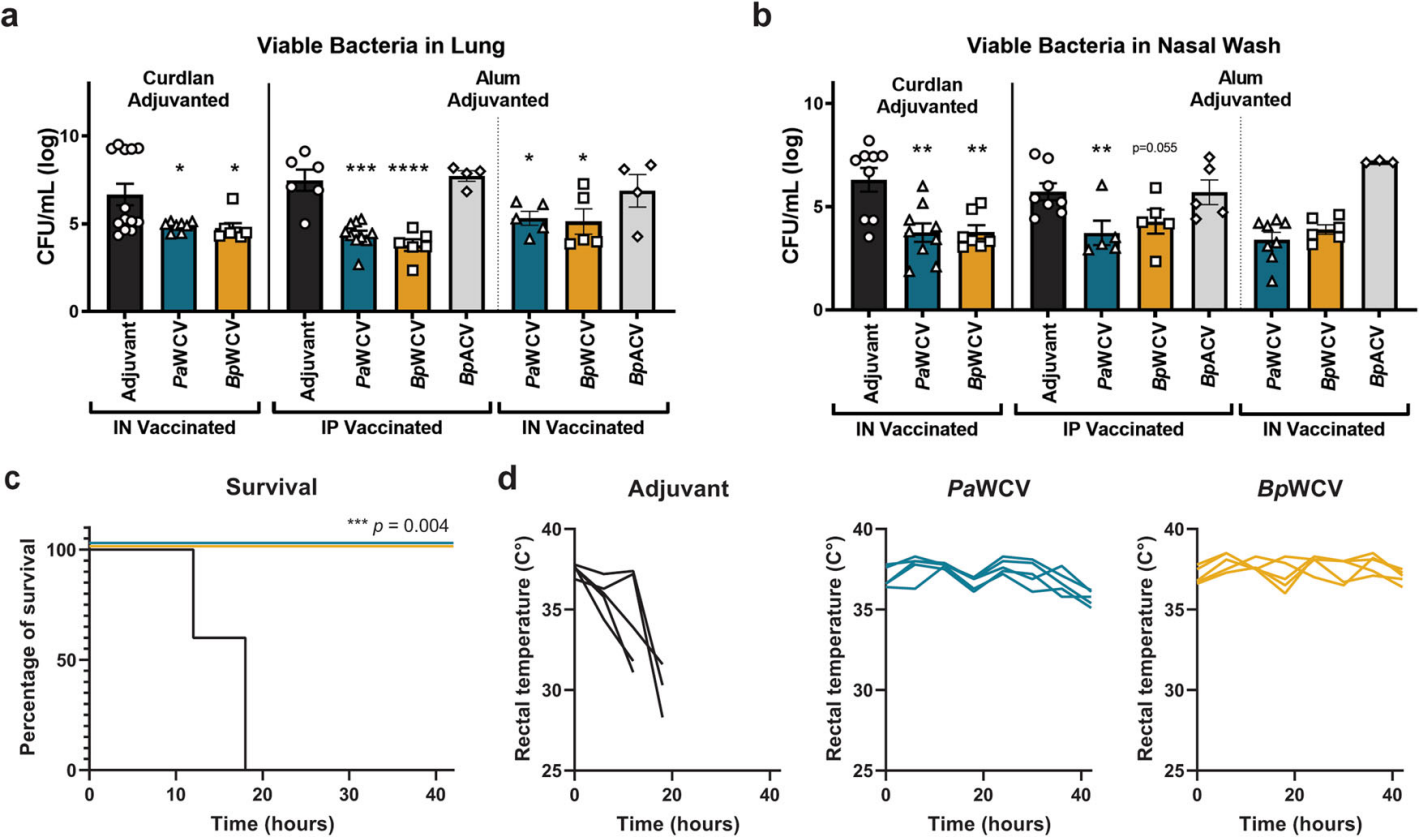
*B. pertussis* whole cell vaccination protects outbred and inbred mice against *P. aeruginosa* acute pneumonia and sepsis. Bacterial burden in the lung (**a**) and the nasal wash (**b**) of mice were vaccinated with *Pa*-WCV, *Bp*-WCV and adjuvant alone as control sixteen hours post challenge in a murine model of pneumonia in outbred CD-1 mice. Each dot represents an individual mouse, and error bars represent standard error of the mean. Experiments in **a** and **b** were performed with *n* = 4–13 mice/group. Experiments in **c** and **d** included *n* = 5 mice/group. The asterisks represent statistical significance determined by ANOVA with a Tukey multiple comparison post-test: *p ≤ 0.05, **p ≤ 0.01, ***p < 0.001, ****p < 0.0001. Survival (**c**) and body temperature (**d**) over time of CD-1 mice vaccinated with *Pa*-WCV, *Bp*-WCV and the alum adjuvant alone as control in a lethal model of sepsis. Log-rank Mantel-Cox analysis of survival.

From our observations that vaccination with *Bp*-WCV leads to the production of antibodies that react with both lab-adapted and clinical *P. aeruginosa* strains, we hypothesized that *Bp-*WCV vaccination is protective against *P. aeruginosa* infection. To test this, outbred mice were immunized as described above and intranasally challenged with *P. aeruginosa* PAO1 34 days post-prime. On day 35, mice were euthanized and the viable bacteria present in the airways were quantified by serial dilution and plating of the lung homogenates and nasal washes. We observed that challenged mice that had been administered the adjuvant alone during the vaccination course had high bacterial burdens in the nares and the lungs one-day post infection (Fig. 2a, b). When adjuvanted with curdlan and immunized intranasally, both *Pa-*WCV and *Bp-*WCV significantly reduced bacterial burden in the lung and nasal wash (Fig. 2a, b). Similar results were observed with alum-adjuvanted vaccines, administered either intranasally or intraperitoneally (Fig. 2a, b). To determine if vaccines provided protection in a sepsis model, CD-1 mice were vaccinated as described above and challenged with a lethal dose of *P. aeruginosa* intraperitoneally. We observed that both *Pa-*WCV and *Bp-*WCV provided significant protection against death (Fig. 2c) and hypothermia (Fig. 2d) in response to a lethal *P. aeruginosa* infection. These findings demonstrate that the cross-reactive response to *P. aeruginosa* induced by *Bp-*WCV immunization is also cross-protective against *P. aeruginosa* pneumonia and sepsis. In the pneumonia model, cross-protection was observed regardless of the adjuvant or route selected for vaccination.

**Fig. 3:**
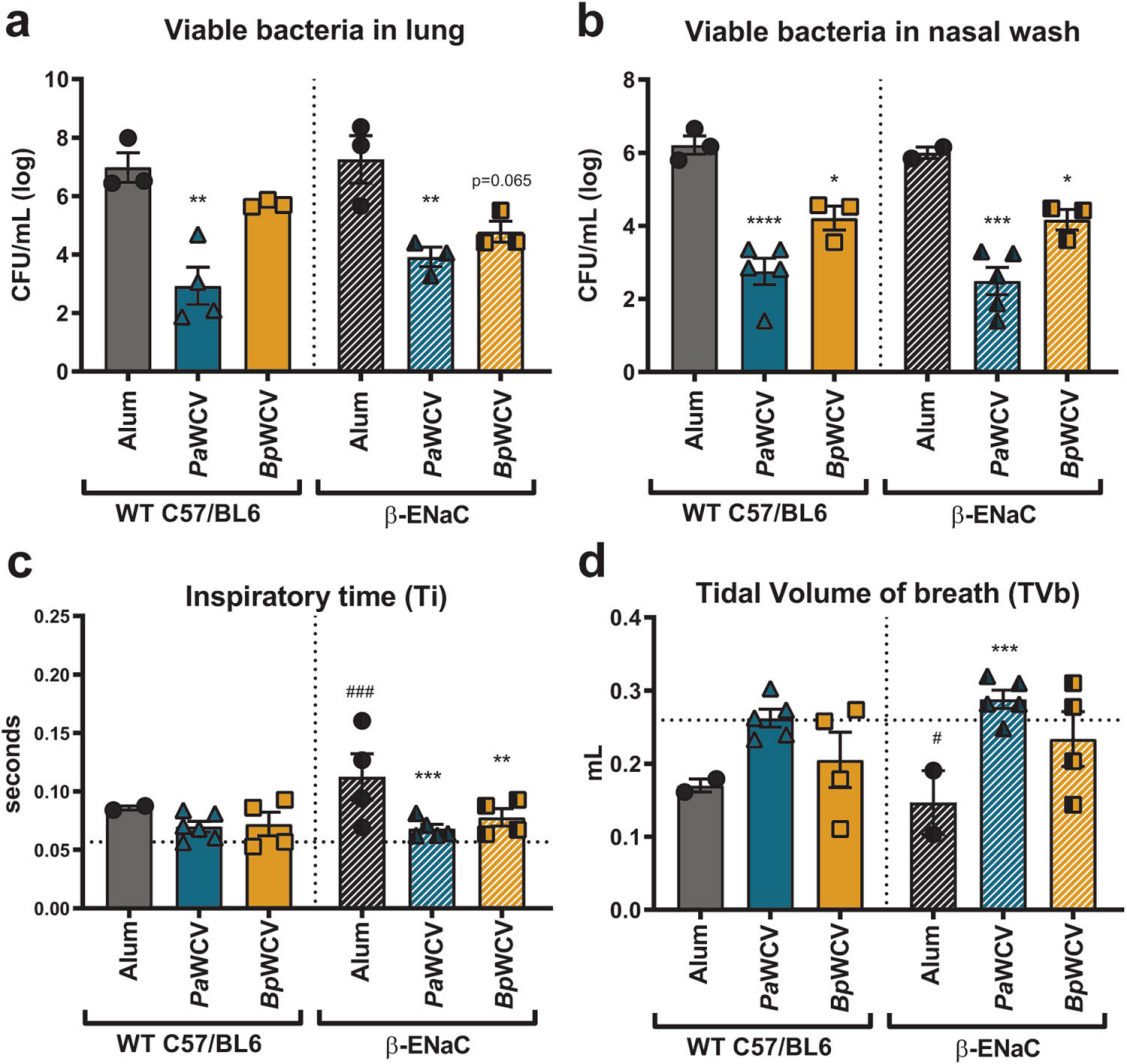
*B. pertussis* whole cell vaccination protects inbred and β-ENaC mice against *P. aeruginosa* acute pneumonia. Intraperitoneal vaccination with *P. aeruginosa* and *B. pertussis* whole cell vaccines adjuvanted with alum decreases bacterial burden in the respiratory tract of C57BL/76 and β-ENaC transgenic mice (**a**, **b**), one-day post infection. Sixteen hours post challenge, groups of mice were dissected and the bacterial burden in the lung (**a**) and nasal wash (**b**) were quantified using serial dilution. Using whole body plethysmography prior to euthanasia, tidal volume of breadth (**c**) and inspiratory time (**d**) were quantified and compared to baseline. Each dot represents an individual mouse, and error bars represent standard error of the mean. Vertical dotted line separates genotypes of mice, horizontal dotted line indicates the average value measured at baseline (**c**, **d**). Experiments were performed with *n* = 3–5 mice/group. In all panels, asterisks over bars indicate comparison to alum-vaccinated control group. In **c** and **d**, hash tags over bars indicate comparison to the baseline value for that group. Statistical significance was determined by ANOVA with a Tukey multiple comparison post-test: *p ≤ 0.05, **p ≤ 0.01, ***p ≤ 0.001, ****p ≤ 0.0001.

*P. aeruginosa* causes difficult-to-treat antibiotic-resistant infections in the lung of patients with the genetic disorder cystic fibrosis (CF)^31,32^. Vaccination against *P. aeruginosa* could be particularly beneficial in these patients to reduce the burden of disease and improve morbidity and mortality associated with *P. aeruginosa* infections. To determine if the observations in outbred CD-1 mice also translate to a CF-like model, we used mice overexpressing the β-ENaC receptor that display CF-like lung phenotypes^33^, and compared them to their C57BL/6 littermates. Mice were vaccinated with *Pa-*WCV, *Bp-*WCV, or the adjuvant alum intraperitoneally on days 0 and 21, and then challenged with *P. aeruginosa* intranasally. Consistent with our findings in the outbred CD-1 mouse model, *Pa-*WCV was able to induce a protective immune response against acute *P. aeruginosa* infection in both C57BL/6 and β-ENaC mice 15 h post-infection (Fig. 3a, b). Interestingly, vaccination with *Bp-*WCV led to a reduction of bacterial burden in the nares but not in the lung in response to *P. aeruginosa* infection. To further characterize the protective response and its impact on respiratory function, we used whole-body plethysmography (WBP). This analysis revealed that the inspiration time (Ti), or time spent on inhaling, was significantly increased in infected β-ENaC transgenic mice, but not C57B/6 mice, compared to the non-challenged baseline (Fig. 3c). Vaccination with either the *Pa-*WCV or *Bp-*WCV was able to resolve this increase and return Ti levels to non-challenged baseline in β-ENaC mice. Furthermore, infection significantly reduced the tidal volume of breadth (TVb) in non-vaccinated β-ENaC mice. We observed that vaccination with either *Pa-*WCV or *Bp-*WCV helped restore baseline TVb (Fig. 3d). No differences in the number of breadths per minute, pause and enhanced pause, tidal mid-expiratory flow (EF50) nor in the time of expiration were observed between any of the treatment groups. Taken together, TVb and Ti data illustrate that *P. aeruginosa* infection led to longer inspiration and shallower breathing in mice, and that both *Pa-*WCV and *Bp-*WCV administration restore this phenotype to baseline (Fig. 3c, d). Consistent with the serology from CD-1 mice, shown in Fig. 1, the *Bp-*WCV was also able to induce anti-PAO1 IgG in the WT and β-ENaC mice (Supplementary Fig. 1).

### *Bp-*WCV cross-reactive antibodies bind GroEL and OprF

**Fig. 4:**
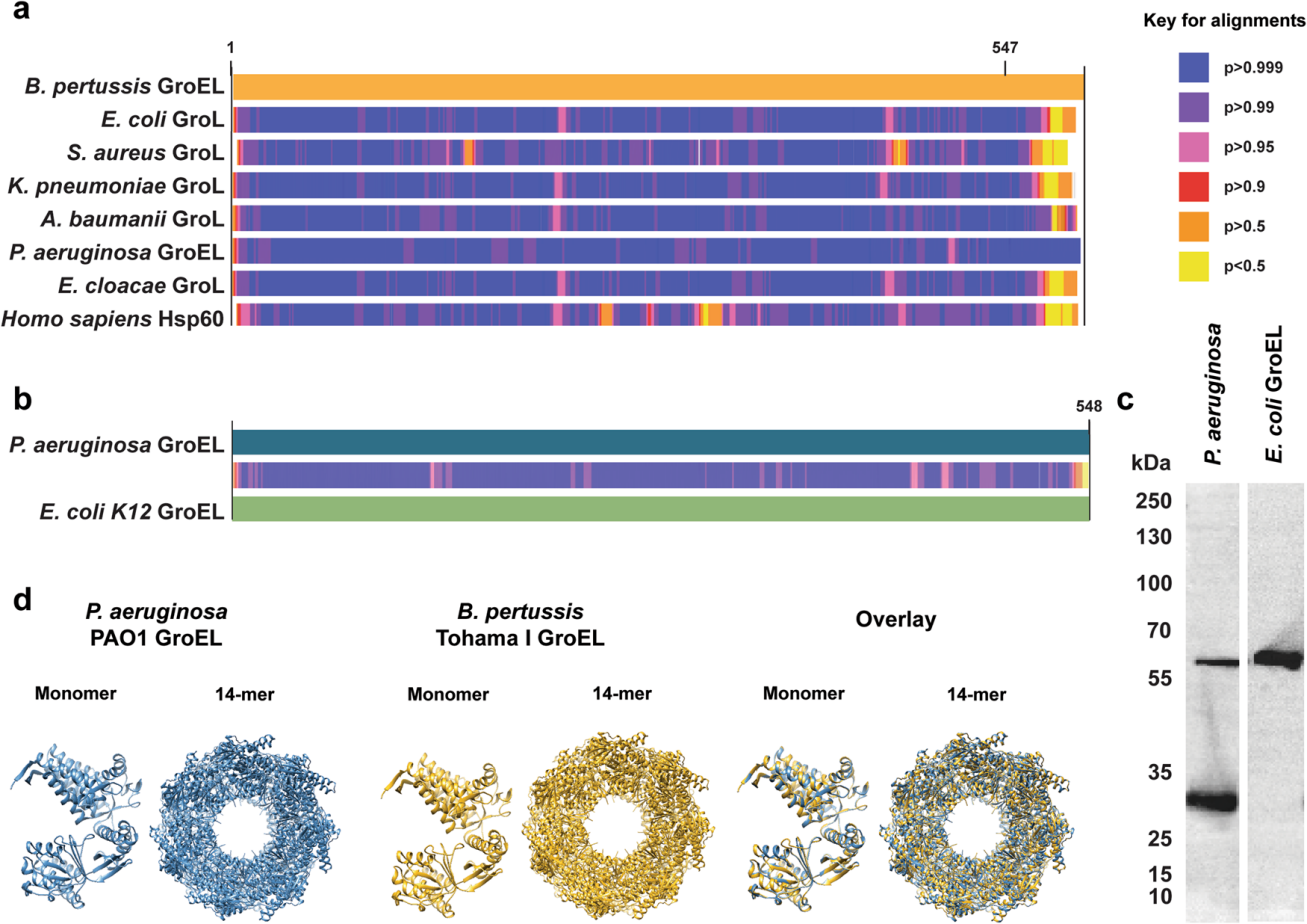
GroEL is a highly conserved bacterial protein, and can be bound by *Bp-*WCV induced serum antibodies. **a** Alignment of *B. pertussis* GroEL amino acid sequence to ESKAPE pathogen homologs and Human hsp60. **b** Alignment of *P. aeruginosa* GroEL to *E. coli* K12 GroEL amino acid sequence. **c** Immunoblotting of *Bp-*WCV serum to *P. aeruginosa* lysate and recombinant *E. coli* GroEL. All blots in this panel were performed in parallel. **d** Structure predictions for *P. aeruginosa* and *B. pertussis* GroEL.

To identify the *P. aeruginosa* antigens bound by antibodies triggered by *Bp-*WCV, we performed immunoprecipitation and mass spectrometry. Immunoprecipitants were formed by incubating the serum with whole *P. aeruginosa* lysate, and purifying antigen-bound antibodies using mass-spectrometry compatible magnetic protein A/G beads. The purified and eluted antigens were analyzed by mass spectrometry, and identified peptides were cross-referenced with the PAO1 protein database (SwissProt TaxID = 208964). This analysis led to the detection of 10 proteins with 2 or more peptides (Supplementary Table 2). The proteins were then sorted based on abundance and percent coverage of the antigen, and the cross-reactive antigens with the highest peptide coverage and abundance were selected for further testing: GroEL (25 peptides, 48% coverage) and OprF (7 peptides, 30% coverage). GroEL is a highly conserved cytosolic chaperonin protein, approximately 60 kDa in size^34^. GroEL is also highly abundant, primarily located in the cytoplasm, but sometimes also embedded in the membrane^34–37^. It is produced by all ESKAPE pathogens, and is relatively highly conserved across these species (Fig. 4). Notably, *B. pertussis* GroEL is also highly similar to the human chaperonin protein *Hsp60* (53.15% identity) (Fig. 4a). GroEL is an essential protein in *P. aeruginosa* and other bacterial species^38^, therefore, no deletion or transposon insertion mutants are available to determine if the cross-reactive antibodies produced in response to *Bp*-WCV bind to this protein. As an alternative, we performed Western blotting with the commercially available *E. coli* GroEL protein, which is highly homologous to the *P. aeruginosa* GroEL (Fig. 4b). We observed that the *Bp-*WCV induced antibodies were capable of binding purified *E. coli* GroEL and that the band had a similar size to one of the antigens detected in *P. aeruginosa* (Fig. 4c, Supplemental Fig. 6). We additionally predicted the structure of these proteins using SWISS-MODEL homology modeling based on the crystalized structure with the highest level of amino acid homology (*Xanthomonas oryzae*; PDB ID: 6KFV) (Fig. 4d) and showed high structural similarity.

**Fig. 5:**
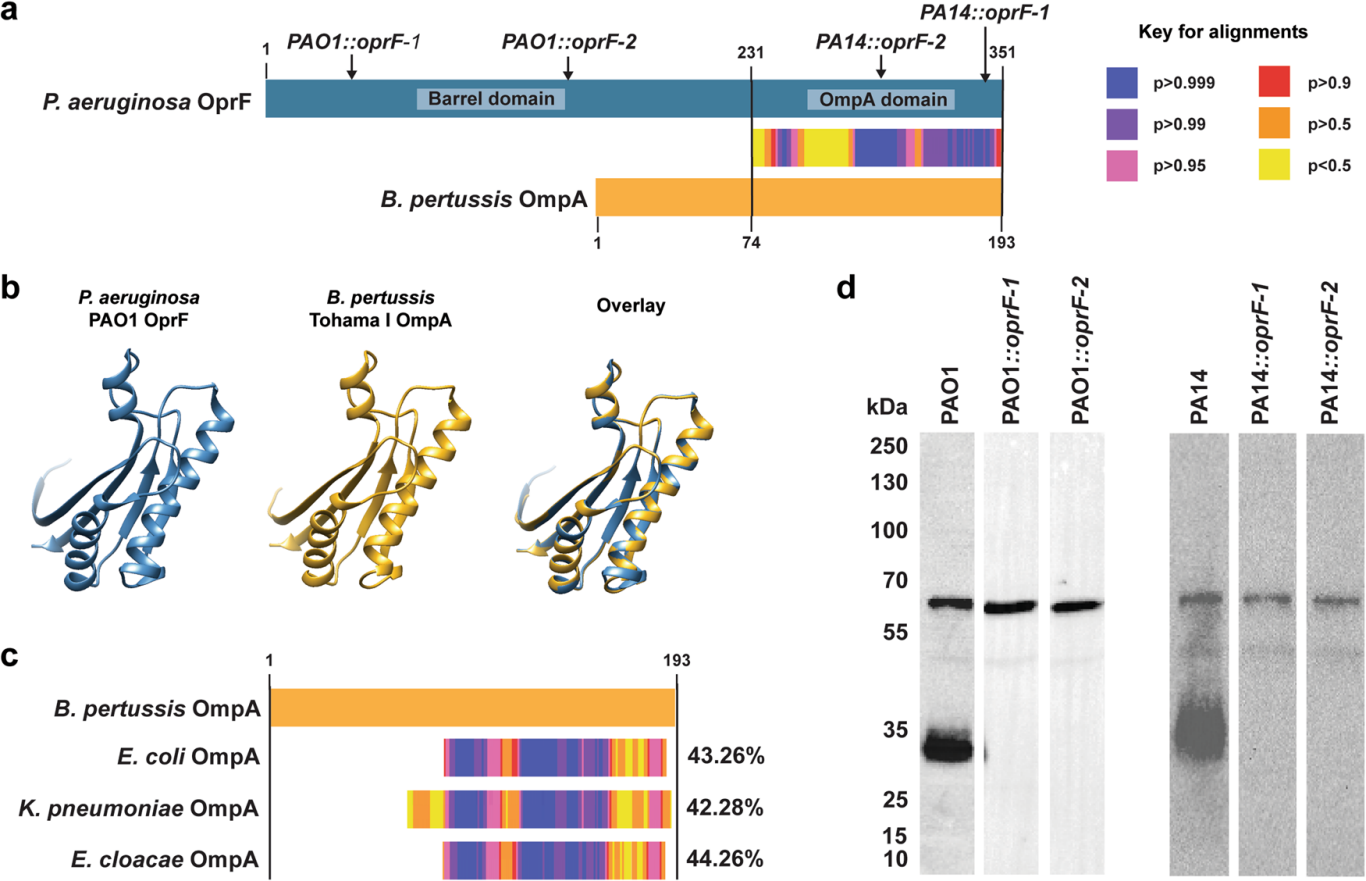
Serum antibodies in *B. pertussis* whole cell immunized mice bind to *P. aeruginosa* OprF, which is homolog to *B. pertussis* OmpA. **a** LAST alignment of *B. pertussis* OmpA amino acid sequence to the *P. aeruginosa* OprF amino acid sequence. **b** Overlay of *P. aeruginosa* OprF OmpA domain and predicted *B. pertussis* OmpA protein. **c** Overlay of *B. pertussis* OmpA sequence to ESKAPE pathogen homologs, found in *E coli*, *K. pneumoniae*, and *E. cloacae*, with percent identity shown to the right. No homologs were identified in *S. aureus*, or *A. baumannii*, or *Homo sapiens*. **d** Western blot analysis of binding of cross-reactive antibodies to wild-type PAO1 and PA14 strains of *P. aeruginosa*, and to transposon mutants containing insertions in the *oprF*, as indicated in part A with an arrow. All blots in this panel were performed in parallel.

The other cross-reactive antigen identified by immunoprecipitation and mass spectrometry is *P. aeruginosa* OprF (PA1777). This protein contains an OmpA-like domain similar to *B. pertussis* OmpA (Fig. 5a, d). Like GroEL, *B. pertussis* OmpA has homologs in other bacteria including *E. coli*, and the ESKAPE pathogens *K. pneumoniae* and *E. cloacae* with over 40% sequence identity (Fig. 5c). To examine binding of *Bp-*WCV induced antibodies to *P. aeruginosa* OprF, we utilized transposon mutants with insertions in the *oprF* gene (Supplementary Table 1). OprF contains an N-terminal β-barrel domain and a C-terminal globular domain (OmpA domain). Both the PAO1 and PA14 ordered transposon mutant libraries contains two mutants with insertions in *oprF*. In PAO1, both insertions are located in the sequence encoding for the N-terminal β-barrel, while both insertions in the PA14 mutants are located in the sequence encoding for the C-terminal domain barrel (Fig. 5a). To test whether insertion in this gene resulted in loss of binding to the smaller antigen identified in Fig. 1c, Western blotting was performed with each parental and mutant strain lysate and the *Bp-*WCV immunized sera. Consistent with our prior observation that the serum from *Bp-*WCV vaccinated mice reacted with a protein of approximately 60 kDa (identified above as GroEL), we observed binding at this size in the transposon mutants and the parental strains (Fig. 5d). In addition, the serum from *Bp-*WCV vaccinated mice also reacted with a protein of approximately 30 kDa in parental PAO1 and PA14 strains, but the binding was not detected against the transposon mutants containing an insertion in the gene *oprF* (Fig. 5d, Supplemental Fig. 7). Taken together, the data obtained via immunoprecipitation, mass spectrometry, and immunoblotting indicate that the *P. aeruginosa* antigens bound by *Bp-*WCV induced antibodies are GroEL and OprF, which both have homologs in *B. pertussis*.

### *B. pertussis* OmpA is a protective antigen for vaccination against *P. aeruginosa*

Our long-term objective is to develop a subunit vaccine against *P. aeruginosa* for at-risk populations, including patients with CF. Given that vaccination with *Bp-*WCV is protective against *P. aeruginosa* and leads to the production of antibodies against *P. aeruginosa* GroEL and OprF, we explored the potential use of these proteins as vaccine antigens. We initiated these studies by determining the level of conservancy of each protein across 130 genome-sequenced clinical *P. aeruginosa* strains from CF patients^39^. We observed that the amino acid sequences of OprF were 100% conserved across these isolates, and GroEL was 100% conserved in all but one clinical isolate, where the GroEL protein contained a single amino acid replacement (V525M). The data obtained suggest that vaccination with these antigens could lead to the production of antibodies that broadly react with clinically relevant *P. aeruginosa* isolates. However, we observed that the amino acid sequence homology of *B. pertussis* GroEL and Hsp60 is also high (Fig. 4a). This suggests that vaccination with *B. pertussis* GroEL could lead to the production of auto-reactive antibodies, which would be undesired. However, *B. pertussis* OmpA does not have a known homolog in humans, so it was selected as the candidate antigen for immunization in this study.

**Fig. 6:**
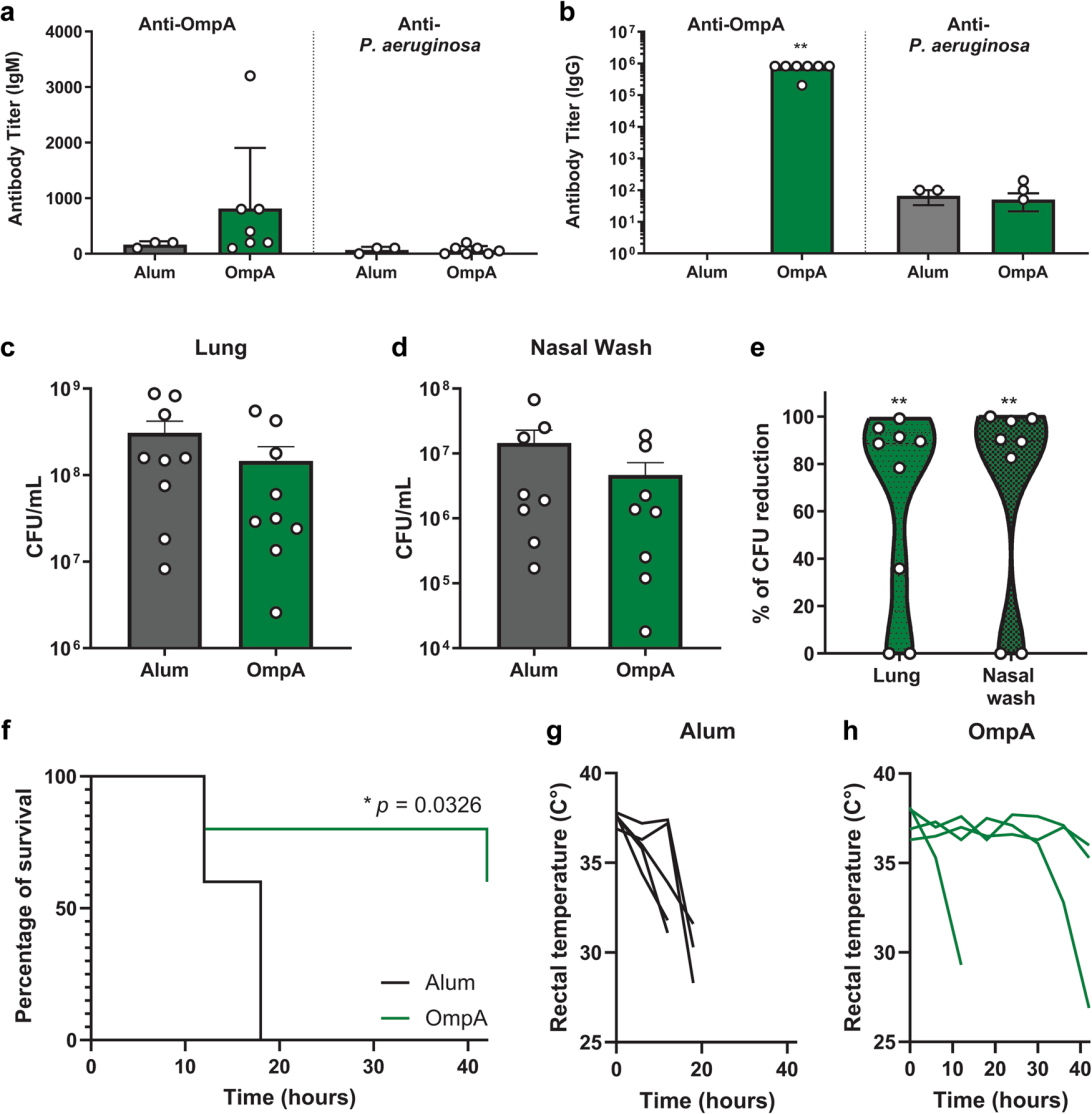
Vaccination with recombinant *B. pertussis* OmpA induces production of cross-reactive antibodies and protection against *P. aeruginosa* in mice. **a**, **b** Anti-*B. pertussis* recombinant OmpA and anti-*P. aeruginosa* IgM and IgG antibody detection by ELISA in the serum of vaccinated mice. Bacterial burden in the lung (**c**) and nares (**d**) and percent reduction in bacterial burden between OmpA and Alum-vaccinated mice (**e**) 15 h post-challenge in a pneumonia model of *P. aeruginosa* infection. Mice survival (**f**) and rectal temperatures (**g** and **h**) over time of mice challenged with *P. aeruginosa* in a lethal model of sepsis. **a**–**e** Experiments were performed with *n* = 7–9 mice/group. In **f**–**h**, experiments were performed with *n* = 5 mice/group. In **a** and **b**, statistical significance determined by ANOVA with a Tukey multiple comparison post-test, while statistical significance was determined in **c**–**e** using a *t*-test: in **a**–**e**, each dot represents an individual mouse, and error bars represent standard error of the mean. Statistical significance in **f** was determined using a Mantel-Cox test. *p < 0.05, **p ≤ 0.01.

To determine if *B. pertussis* OmpA is a protective antigen for immunization against *P. aeruginosa*, we vaccinated mice intraperitoneally with 35 μg of purified recombinant *B. pertussis* OmpA protein produced in *E. coli* and formulated with the adjuvant alum. Mice received a boost with the same formulation on day 21, and were challenged with *P. aeruginosa* PAO1 on day 34. The following day, animals were euthanized and CFUs in the respiratory tract were quantified. We first determined whether recombinant *B. pertussis* OmpA was antigenic, by examining the production of anti-*B. pertussis* OmpA IgG following vaccination and boost (Fig. 6a, b). We observed that the *B. pertussis* OmpA immunized animals produced anti-*B. pertussis* OmpA IgG serum antibodies 34 days post initial vaccination (Fig. 6b). Anti-*B. pertussis* OmpA IgM levels were detectable, but not significantly greater than the control group. Interestingly, we did not detect a significant production of anti-*P. aeruginosa* antibodies in response to vaccination with *B. pertussis* OmpA (Fig. 6b). Mice were then challenged intranasally with *P. aeruginosa* and bacterial burden was determined approximately 15 h post-challenge. While the bacterial loads in the lung and the nares of mice challenged with *P. aeruginosa* were not statistically different between each vaccine group (Fig. 6c, d), vaccination with *B. pertussis* OmpA led to the reduction of bacterial burdens by 64.2% in the lung and 69.9% in the nares (Fig. 6e). To determine if these differences were biologically relevant during infection, we used a more sensitive murine model of *P. aeruginosa* infection: the sepsis model. Mice were vaccinated as described above and challenged intraperitoneally with a lethal dose of *P. aeruginosa*. Survival as well as rectal temperature were then followed for the subsequent days. We observed that in that model, vaccination with *B. pertussis* OmpA significantly protects mice against death (Fig. 6f). While all control mice died from infection within the first 20 h and displayed temperature loss, mice vaccinated with OmpA survived longer and only two died from infection, one at 12, and one at 42 h post-challenge. These data together illustrate that vaccination with *B. pertussis* OmpA is sufficient to protect against *P. aeruginosa* infection.

## Discussion

In this report, we sought to determine the protective role of a *B. pertussis* whole cell vaccine against the Gram-negative pathogen *P. aeruginosa*. We observed that *Bp-*WCV led to a decrease in *P. aeruginosa* PAO1 bacterial burden in the airways of CD-1, C57BL/6, and transgenic β-ENaC mice. We further demonstrated that *Bp-*WCV was able to induce the production of anti-*P. aeruginosa* IgG, and that these antibodies bind *P. aeruginosa* GroEL and OprF. These proteins have homologs in *Bordetella pertussis* that we hypothesize are inducing cross-protection: GroEL and OmpA. Furthermore, we demonstrated that these antigens are conserved in clinical isolates of *P. aeruginosa*, and that antibodies produced against *B. pertussis* also recognize clinical *P. aeruginosa* strains. Finally, we tested recombinantly produced *B. pertussis* OmpA protein as antigen for immunization against *P. aeruginosa*, and observed that it was antigenic and contributed to protection against *P. aeruginosa* pneumonia and sepsis in mice.

The antigens identified in this study, GroEL and OprF, are known to be antigenic proteins of *P. aeruginosa*. GroEL, which works in conjunction with the protein GroES to form a well-characterized barrel-shaped chaperonin, is conserved amongst many bacterial species^40–43^. This bacterial protein is also highly abundant and immunogenic^34,35^. GroEL is a known immunogenic protein of *B. pertussis* and the presence of circulating anti-GroEL antibodies is characteristic of patients vaccinated with *B. pertussis* whole cell vaccines^44^. While some studies have shown that GroEL from various species can be protective as vaccine antigen and adjuvant in pre-clinical studies, GroEL from *B. pertussis* failed to protect against infections caused by this microorganism in mice^45,46^. The efficacy of GroEL may be in part limited by the fact that this protein is primarily expressed in the cytosol, although in some cases it can be surface-exposed^46,47^. We observed that GroEL is highly conserved amongst Gram-negative bacteria of clinical relevance. In addition, it is known that GroEL is similar to the mitochondrial heat shock protein Hsp60 (51.15% identity of Hsp60 to *P. aeruginosa* GroEL). As a result, we speculate that the whole GroEL protein may not be a viable candidate vaccine antigen for vaccination in humans due to potential undesired cross-reactivity^34^. For example, an increase in anti-*P. aeruginosa* GroEL antibody titers is observed in cystic fibrosis proceeding the onset of diabetes, and is hypothesized to play a role in the mitochondrial stress associated with diabetes^34^. A similar phenomenon has been observed in the context of *Chlamydia pneumoniae* infection, which is associated with increased titers of anti-Hsp60 IgG that correlate to myocardial infarction and coronary death following infection^48,49^. If GroEL is a protective antigen for *P. aeruginosa*, it would be necessary to further analyze the epitopes recognized by the *Bp-*WCV induced cross-reactive antibodies to identify specie-specific epitopes and reduce potential off-target effects with Hsp60.

The other cross-reactive antigen identified in this study, OprF, is a highly abundant outer membrane porin. OprF also plays significant roles in pathogenesis of *P. aeruginosa*^49,50^. OprF is capable of transporting ions and low molecular-mass carbohydrates, such as sodium chloride, glucose, and sucrose^51^. OprF is vital in maintenance of the cell membrane and regulation of cell morphology, quorum sensing, and virulence factors including adhesion to eukaryotic cells, biofilm production, and type 3 secretion system^50,52–55^. Deletion or functional knockout of the OprF gene in *Pseudomonas aeruginosa* leads to decreased virulence in several infection models, including *Caenorhabditis elegans*, cell culture, and mouse models^50,56^. OprF is also overexpressed in anaerobic conditions, such as those mimicking CF-like lung phenotypes^57^. OprF is a known immunogenic protein of *P. aeruginosa* and CF patients with history of *P. aeruginosa* infection have circulating anti-OprF antibodies^58^. OprF has been extensively characterized as antigen through both pre-clinical and clinical studies^59,60^. One study found that dendritic cells pulsed with OprF induce a protective response following adoptive transfer^61^. Another group found that two peptides, linear epitopes from the OmpA domain of *P. aeruginosa* OprF, conjugated to the carrier protein KLH were able to induce a protective response in mice^62^. A recombinant OprF-OprI vaccine was tested in humans, and induced strong IgG and IgA antibody response^59^. *P. aeruginosa* OprF contains two domains, an N-terminal membrane embedded 8-fold β-barrel porin, and a C-terminal globular domain known as the OmpA domain. While the studies referenced above were focused primarily on the beta-barrel domain of OprF, the structure and sequence homology data obtained in this study seem to indicate that cross-reactivity is potentially mediated via the globular OmpA domain of OprF. OmpA*-*family proteins are produced by a wide variety of pathogenic and nonpathogenic bacteria, including *Haemophilus influenzae, Klebsiella pneumoniae*, and *Chlamydia trachomatis*, but not all species produce both domains found in the *P. aeruginosa* protein^49,63^. Further investigation, such as epitope mapping, could be used to identify the protective antigens for subsequent subunit vaccine formulation. Notably, we observed 100% conservation of the OprF amino acid sequence in clinical isolates of *P. aeruginosa*, suggesting that it could be a viable antigen for protection against a variety of strains of the pathogen. In addition, *P. aeruginosa* produces several OmpA family proteins that could likely be used for vaccination against this bacterium^64,65^. By examining the conserved epitopes of OmpA domains, it may be possible to target multiple protein antigens or pathogens simultaneously. In this study, we observed that vaccination with *B. pertussis* OmpA help reduce bacterial burden in both the lung and the nasal wash (Fig. 6e) in a pneumonia model and was sufficient to significantly protect from death in a sepsis model in mice (Fig. 6f–h). These results were observed despite the lack of detection of antibodies that bind *P. aeruginosa*. A similar phenomenon was observed in mice vaccinated with the live-attenuated BPZE1 *B. pertussis* vaccine, were the vaccine protected against virulent influenza A viruses in the absence of cross-reactive antibodies^66^. A similar study highlighting the role of BPZE1 in protection against lethal pneumococcal disease demonstrated that protection was lost in MyD88 knock out mice^24^. Our work, together with these two additional examples of cross-protection against other pathogens mediated by whole cell *B. pertussis* vaccines, suggest that the mechanism of action is independent of antibody production and potentially relies on T cells or trained immunity. In addition, protection provided by vaccination with OmpA was inferior to the protection provided by *Bp*-WCV. This also suggests that other cross-reactive antigens such as GroEL could play a role in protection against *P. aeruginosa*. Interestingly, vaccination with *B. pertussis* OmpA was not protective against *B. pertussis* challenge in outbred CD-1 mice (Fig. S2). While vaccination with recombinant *B. pertussis* OmpA led to the production of anti-OmpA antibodies, no anti-*B. pertussis* antibodies were generated in response to OmpA vaccination. We hypothesize that this result is likely due to poor folding of this membrane protein during purification (present in inclusion bodies in *E. coli*). Altogether, these data suggest that additional work is needed to refine the sequence and improve the immunogenicity of OmpA for vaccination. We also propose that this antigen should be used in combination with other surface antigens or virulence factors to provide a stronger protective response against *P. aeruginosa*.

This work highlights that the non-specific effects of *B. pertussis* whole cell, but not acellular vaccination, could also provide protection against the respiratory pathogen *P. aeruginosa*. During the last thirty years, whole cell pertussis vaccination has been replaced by acellular immunization in many countries, including the US. It is currently unknown whether protection against *P. aeruginosa* mediated by *Bp-*WCV occurs in the human population. As the acellular-only vaccinated population continues to age, follow-up studies examining the incidence of *P. aeruginosa* infection in *Bp-*WCV and *Bp-*ACV populations will become feasible and help shed light on the potential clinical impact of our observations. In addition, future studies focused on vaccination using the *B. pertussis* OmpA protein could be used to identify the epitopes involved in cross-reactivity.

Taken altogether, this work illustrates that cross-reactivity amongst antigens in vaccines and unrelated pathogens could be utilized as a source for antigen identification, and exemplifies a mechanism of the non-specific effects of a *B. pertussis* whole cell vaccine. Further research is necessary to determine if this type of strategy could be applied to other bacterial pathogens with structurally related proteins, such as some of the ESKAPE pathogens.

## Methods

### Bacterial strains and growth

*P. aeruginosa* strain PAO1 Vasil^67^ was used for vaccine preparation and murine challenges. For assays using transposon mutants, we utilized the University of Washington PAO1 transposon mutant library^68^. To prepare a challenge dose of *P. aeruginosa*, PAO1 was grown on the Miller formulation of lysogeny agar (LA-Miller formulation) overnight at 37 °C. A single colony was used to start 3 mL cultures, which were incubated while shaking, overnight at 37 °C. To prepare a culture in exponential phase, aliquots of the overnight culture were diluted 1:100 in fresh Miller formulation of lysogeny broth (LB-Miller), allowed to grow for approximate 4–5 h, then centrifuged, resuspended in sterile phosphate-buffered saline (PBS), and diluted to the desired dose using optical density (OD_600_; SpectraMax i3 and Cerillo Stratus plate readers). The dose was quantified using serial dilution and plating on Difco Pseudomonas isolation agar (PIA; Becton, Dickinson and Company, Cat #292710).

For ELISA and Western blots, PAO1 transposon mutant and parental strains were struck on LA-Miller supplemented with tetracycline (50 µg/mL; Sigma-Aldrich) (Supplementary Table 1). Clinical isolates of *P. aeruginosa*, described by Burns et al.^39^, were kindly provided by Dr. Robert Ernst (Supplementary Table 2). Strains were grown on PIA overnight at 37 °C before being swabbed, resuspended in PBS, and used for analysis. Serotypes of clinical isolates were predicted using the *Pseudomonas aeruginosa* serotyper (PAst)^69^. Other bacterial species used for western blot analysis, including *Escherichia coli, Staphylococcus aureus, Klebsiella pneumoniae, Acinetobacter baumannii*, and *Enterobacter cloacae* were grown overnight on LA at 37 °C (Supplementary Table 1).

### Vaccine preparation

*Pseudomonas aeruginosa* whole cell vaccine (*Pa-*WCV) was prepared as described previously in Blackwood et al.^29^. Briefly, *P. aeruginosa* PAO1 Vasil was grown on PIA overnight, swabbed from the Petri dish into sterile, endotoxin-free PBS, diluted, and heat-killed by incubation for 1 h at 60 °C. For vaccination against *B. pertussis*, we used the National Institute for Biological Standards and Control (NIBSC 94/532, Batch 41S) as whole cell vaccine, and Infanrix (GlaxoSmithKline) as a acellular vaccine, each diluted to 1/50th of the human dose. Vaccines were formulated to include 20 µL of the killed bacterial suspension, and either 200 µg of the adjuvant curdlan or 62.5 µg of the adjuvant alum, in a total volume of 40 µL if administered intranasally, 50 µL if administered intramuscularly or intraperitoneally. Curdlan (Beta-1,3-glucan from *Alcaligenes faecalis*, Invivogen, #54724-00-4) was prepared at a concentration of 200 µg in 20 µL volume, and mixed into the vaccine immediately before administration^29,30,70,71^. Alum-adjuvanted vaccines were allowed to adsorb for 1 h at room temperature, with rotation, and were thoroughly mixed immediately prior to administration to animals. *B. pertussis* OmpA was produced by GenScript based on the full-length sequence of OmpA from the *B. pertussis* strain Tohama I and purified by the vendor using a His6 tag on the C-terminus of the protein. Purity of recombinant *B. pertussis* OmpA was greater than 90% as determined by SDS-PAGE under reducing conditions and endotoxin contents were lower than 0.4 EU/mg (data provided by the manufacturer). Recombinant OmpA was formulated with alum and administered at a dose of 35 µg/mouse.

### Murine immunization and challenge models

To examine vaccine efficacy, we first used an out-bred mouse model with groups of 6-week-old CD-1 female mice (Charles River Strain 022). We then utilized a groups of transgenic B6.Cg-Tg(Scgb1a1-Scnn1b) (β-ENaC) mice (kindly provided by Dr. Livraghi-Butrico, University of North Carolina) in comparison to wild-type (WT) C57BL/6 littermates^72^. For these studies, 13–15-week-old female mice, age matched per group, were used. All animal care and use were in compliance with the National Institutes of Health Guide for the Care and Use of Laboratory animals. The animal protocols used in this study were approved by the West Virginia University Institutional Animal Care and Use Committee (WVU-ACUC protocol 1606003173).

Vaccines described above were administered either intranasally under anesthesia with ketamine (77 mg/kg) (Patterson Veterinary #07-803-6637) and xylazine (7.7 mg/kg) (Patterson Veterinary #07-808-1939) in 0.9% saline^29^, or intraperitoneally (IP), and compared to adjuvant-only vaccination. Vaccines were administered to mice on days 0 and 21.

For the pneumonia model, thirty-four days post initial vaccination, mice were anesthetized as described above, and infected intranasally with 20 µL of bacterial culture containing 3–5 × 10^7^ CFU PAO1. Approximately 15 h post-infection, mice were euthanized by IP injection of Euthasol® Euthanasia solution C IIIN (390 mg pentobarbital/kg) (Patterson Veterinary #07-805-9296) in 0.9% NaCl. Following euthanasia, blood was collected via cardiac puncture. Lung and spleen were aseptically removed and weighed prior to any further treatment or analysis. Nasal wash was collected by flushing 1 mL of sterile PBS through the nasal cavity.

Bacteria were quantified in the lung and nasal wash. The nasal wash was serially diluted and plated on PIA, then grown overnight at 37 °C. The lung was homogenized using a Polytron PT 2500 E homogenizer (Kinematica), then serially diluted, plated, and incubated overnight at 37 °C before colony counting.

For the sepsis infection model, thirty-four days after the initial vaccination, mice were infected intraperitoneally with a dose of 3 × 10^6^ CFU PAO1. Mice were scored for morbidity every six hours post challenge during 96 h to monitor infection progress, attending to six different criteria: (1) appearance, (2) activity, (3) eyes closure, (4) respiration quality, (5) temperature loss and 6) body weight loss. Each variable was given a score between 0 and 4 (0 = no symptoms – 4 = worst symptoms). Mice reaching a score of 4 in any of the variables or an accumulative score of 15 or above were euthanized following protocols approved by West Virginia University institutional animal use and care committee.

### Serology

Antibody titers were quantified using an enzyme-linked immunosorbent assay (ELISA). The 96-well microtiter plates were coated with 50 µL of PBS containing 2 × 10^7^ CFU grown overnight on PIA. Following coating, plates were washed thrice with PBS + 0.05% Tween 20 (Thermo Fisher Scientific, #BP337-500) (PBS-T), then blocked using 2% weight/volume (w/v) bovine serum albumin (BSA) (Research Products International, #A30075) overnight at 4 °C. Serum samples were prepared at a dilution of 1:50 in 2% w/v BSA, then diluted serially down the plate to a maximum dilution of 1:819200, and incubated overnight at 4 °C. Following incubation with the serum, plates were washed four times with PBS-T. Anti-IgG secondary antibody conjugated to alkaline phosphatase (SouthernBiotech, #1030-04) was diluted 1:2000 in 2% w/v BSA, and 100 µL were added to each well, then incubated at 37 °C for one hour. Plates were washed 5 times with PBS-T, then incubated with Pierce p-Nitrophenyl Phosphate (PNPP) (Thermo Fisher Scientific, #34045) for 30 min, per the manufacturer’s instructions. OD_405_ was quantified using SpectraMax i3 (Molecular Devices LLC). Titer was quantified by calculating the highest dilution at which the OD_405_ signal was double that of the blank, and any samples for which no signal was detected were assigned a value of 1 for statistical analysis^29,71^.

### Whole body plethysmography

To examine the impact of infection, with or without prior vaccination, on the breathing and respiratory function of animals, we studied WT and β-ENaC transgenic mice in a Buxco Small Animal Whole Body Plethysmography (WBP) chamber (Data Sciences International). Animals were acclimated to the chamber during a 20-min session in the week prior to the recorded WBP sessions. A baseline measurement of breathing was recorded the day prior to infection, and then post-infection breathing was monitored immediately prior to euthanasia, 15 h post-infection. All WBP sessions included 5 min of acclimation, then data were recorded for 15 min. Data were compiled, analyzed using the FinePointe software (Data Sciences International), and then exported to GraphPad Prism (Version 7; GraphPad Software, Inc.) for statistical analysis. Collected data include breadths per minute, enhanced pause, pause, tidal midexpiratory flow (EF50), tidal volume of breadth (TVb), time of inspiration (Ti), and time of expiration (Te).

### Immunoblotting

To further examine the binding of antibodies in the polyclonal serum, we performed western blot analyses using serum from vaccinated or non-vaccinated groups against bacterial lysates and purified proteins. Bacteria of interest were grown as described above, swabbed into sterile PBS, lysed using sonication, and the protein concentration quantified using bicinchoninic acid assay (BCA) (Thermo Fisher, #23225). Outer membrane proteins were isolated using sodium lauroyl sarcosinate as described by Hernandez-Alles et al.^73^. Unless otherwise designated, 10 µg of protein were added to Laemmli buffer (Bio-Rad, #1610747) + 355 mM β-mercaptoethanol (Thermo Fisher, #21985023) and boiled at 95 °C for 5 min. Samples were loaded into Invitrogen Novex WedgeWell 10% Tris-Glycine protein gel (ThermoFisher Scientific #XP00100PK2) and resolved by gel electrophoresis. Proteins were then transferred using a wet-transfer method onto rehydrated Immun-Blot® PVDF Membrane (Bio-Rad, #1620177), and the membranes were blocked overnight in 5% w/v skim milk (Nestle Carnation, # 000500002292840) in PBS-T at 4 °C. Next, membranes were treated with pooled murine serum samples, at a concentration of 1:5000 in 1% w/v skim milk in PBS, for two hours at room temperature, with orbital shaking. The membranes were washed 3 times with PBS-T then treated with anti-IgG secondary antibody conjugated to horseradish peroxidase (HRP) (Novus Biologicals, # NBP1-75130) at a 1:5000 concentration in 1% w/v skim milk in PBS, for 1 h at room temperature with orbital shaking. The membranes were washed again 4 times in PBS-T and developed using SuperSignal West Femto Maximum Sensitivity Substrate (Thermo Scientific, #34095). Chemiluminescence signal was detected using a Chemidoc Touch Imaging System (Bio-Rad, #1708370). For analysis, the chemiluminescence images were overlaid onto colorimetric images of the ladder, visualized, and analyzed using Image Lab software version 6.1 (Bio-Rad Laboratories). All blots shown within a figure were derived from the same experiment and/or processed in parallel, imaged together, and aligned using the ladder.

### Immunoprecipitation and mass spectrometry

*P. aeruginosa* PAO1 was grown as described above, and 750 µg of PAO1 culture lysate was incubated overnight with pooled *Bp-*WCV serum. Immunoprecipitation was performed using the Pierce MS-Compatible Magnetic IP Kit and Protein A/G beads, per the manufacturer’s instructions (ThermoFisher Scientific, #90409). The resulting purified immunoprecipitation products were analyzed by Liquid chromatography–mass spectrometry in the WVU Mass Spectrometry CORE Facility, according to the protocol in Shevchenko et al.^74^. Data were acquired on a Thermo Q Extractive mass-spectrometer, and peptides identified were mapped using Proteome Discoverer software version 2.3.0.523, using the SeQuestHT algorithm. The database used for mapping was *P. aeruginosa* PAO1 SwissProt TaxID=208964. Only peptides of high confidence designation by SeQuestHT were considered.

### Protein structure modeling

Proteins of interest were visualized in UCSF Chimera software^75^. Protein structures for *P. aeruginosa* OprF (PDB ID: 4RLC) and OmpA (PDB ID: 5U1H) were downloaded from Protein Data Bank (PDB)^76^. Homology modelling of *B. pertussis* OmpA was performed using SWISS-MODEL homology modeling server^77^, and the highest homology protein was selected for reference (PDB ID: 5U1H). Both GroEL structures depicted were modelled similarly using SWISS-MODEL and based on GroEL from *Xanthomonas oryzae* (PDB ID: 6KFV). In comparing *P. aeruginosa* and *X. oryzae* protein sequences, there was a 78.05% sequence identity, and the model had a QMQE score of 0.80 and QMEAN of −0.15. Similarly, the *B. pertussis* GroEL had 75.19% identity with the *X. oryzae* protein sequence, and the model had a QMQE score of 0.79 and QMEAN of −0.67. Structures were visualized and overlaid using the MatchMaker structure analysis tool in UCSF Chimera software^75^.

### Statistical analysis

The statistical analysis of the data presented here was performed using the software Prism version 8 (GraphPad). For experiments performed with mice, each datapoint represents the value for a single individual animal. Three distinct technical replicate measurements were performed for each mouse, and standard error of the mean were calculated. Comparisons between two groups were performed using an unpaired Student’s *t*-test. Comparisons between three or more groups were analyzed by one-way analysis of variance (ANOVA). When applicable, a post-ANOVA Tukey’s multiple comparison test was used for data following a normal distribution. Given the small sample size, population distributions were assumed to follow a normal distribution. Survival curves were compared using a Log-rank Mantel-Cox test.

### Reporting summary

Further information on research design is available in the Nature Research Reporting Summary linked to this article.

## Supplementary information

Suppmental Information

REPORTING SUMMARY

## Data Availability

The authors confirm that the data supporting the findings of this study are available within the article. Any additional information necessary to replicate and build upon the methods or findings reported in the article is available upon request to the corresponding author.
