# Supplementary material for: Bordetella pertussis whole cell immunization protects against Pseudomonas aeruginosa infections
Source: NPJ Vaccines. 2022 Nov 10;7:143. doi: 10.1038/s41541-022-00562-1 (PMC9649022; doi:10.1038/s41541-022-00562-1)
Supplement: Supplementary file 1 — Suppmental Information [file 41541_2022_562_MOESM1_ESM.pdf]

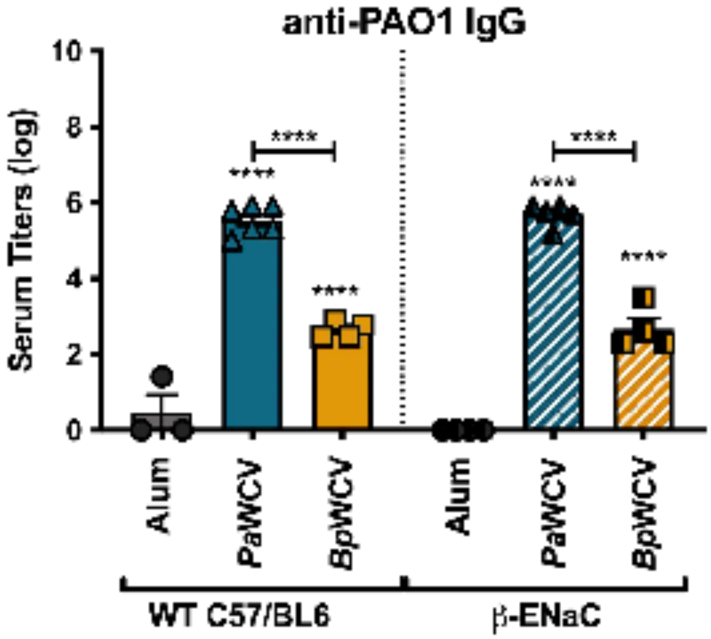

Supplementary Figure 1. Intraperitoneal injections of *P. aeruginosa* and *B. pertussis* whole cell vaccines induce anti-*P. aeruginosa* antibody production in WT C57/BL6 and β-ENaC transgenic mice. Serum titers detected by ELISA using whole *P. aeruginosa* PAO1 bacteria as antigens. Experiments were performed with n=3-6 per group. Each dot represents an individual mouse, and error bars represent standard error of the mean. Asterisks represent statistical significance determined by one-way ANOVA. ****$p<0.0001$

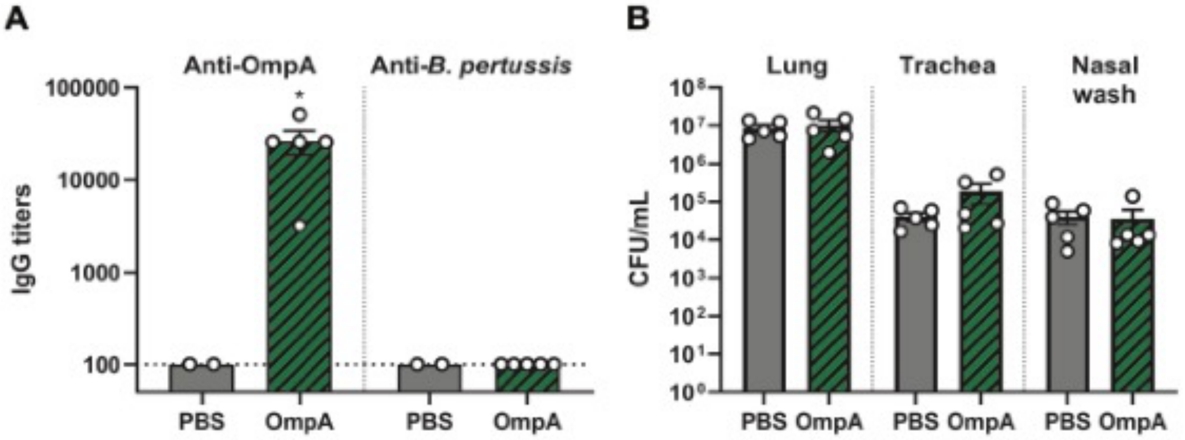

Supplementary Figure 2. Vaccination with *B. pertussis* OmpA does not protect mice against *B. pertussis* challenge. A. ELISA determination of IgG titers against *B. pertussis* OmpA and bacterial cells at day 34 post-vaccination. B. Bacterial burden in the lung, trachea, and nasal wash of mice vaccinated with *B. pertussis* OmpA three days post-challenge. Experiments were performed with an n=5 mice/group. Each dot represents an individual mouse, and error bars represent standard error of the mean. Asterisks represent statistical significance determined by one-way ANOVA. * $p < 0.05$

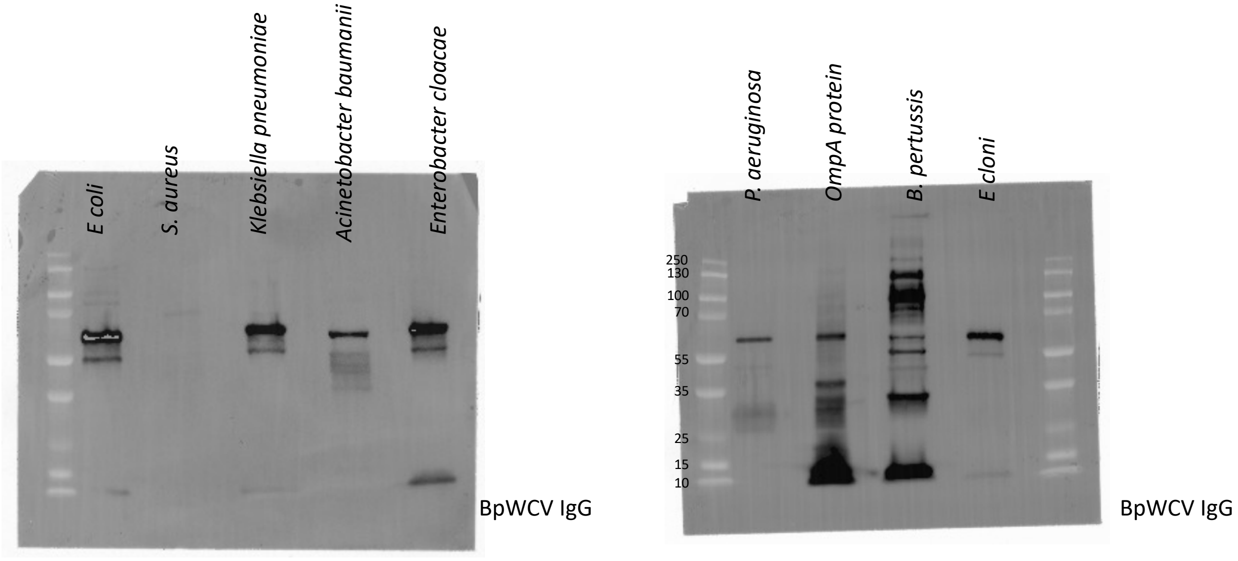

Supplemental Figure 3. Antibodies produced following *B. pertussis* whole cell immunization produce cross-reactive antibodies. Western blot using *Bp*-WCV immunized sera against 10 µg of whole cell *B. pertussis*, *E. coli, S. aureus, K. pneumoniae, A. baumannii, P. aeruginosa, and E. cloacae* bacterial lysates. All blots in this panel were performed in parallel.

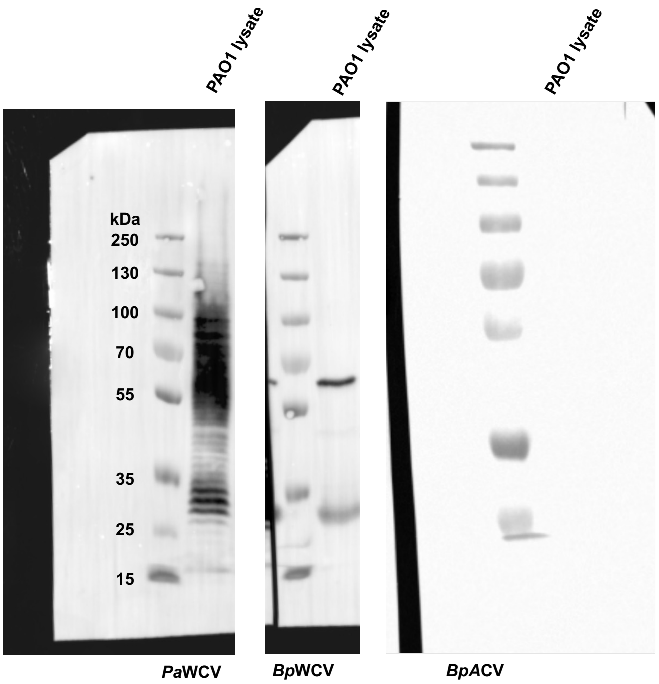

Supplemental Figure 4. Antibodies produced following *B. pertussis* whole cell immunization produce cross-reactive antibodies. Western blot against *P. aeruginosa* bacterial lysate using *Pa*-WCV, *Bp*-WCV , or *Bp*-ACV sera from pooled IN and IP immunized mice.

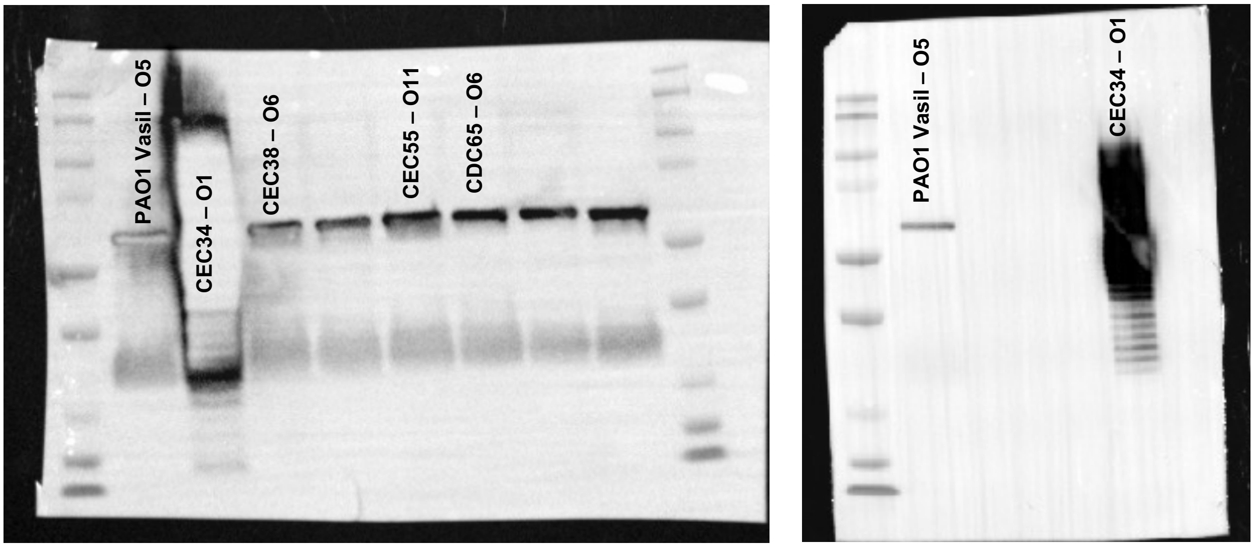

Supplemental Figure 5. Antibodies produced following *B. pertussis* whole cell immunization produce cross-reactive antibodies. Western blot against *P. aeruginosa* clinical isolate bacterial lysate using pooled *Bp*-WCV immunized sera. All blots in this panel were performed in parallel.

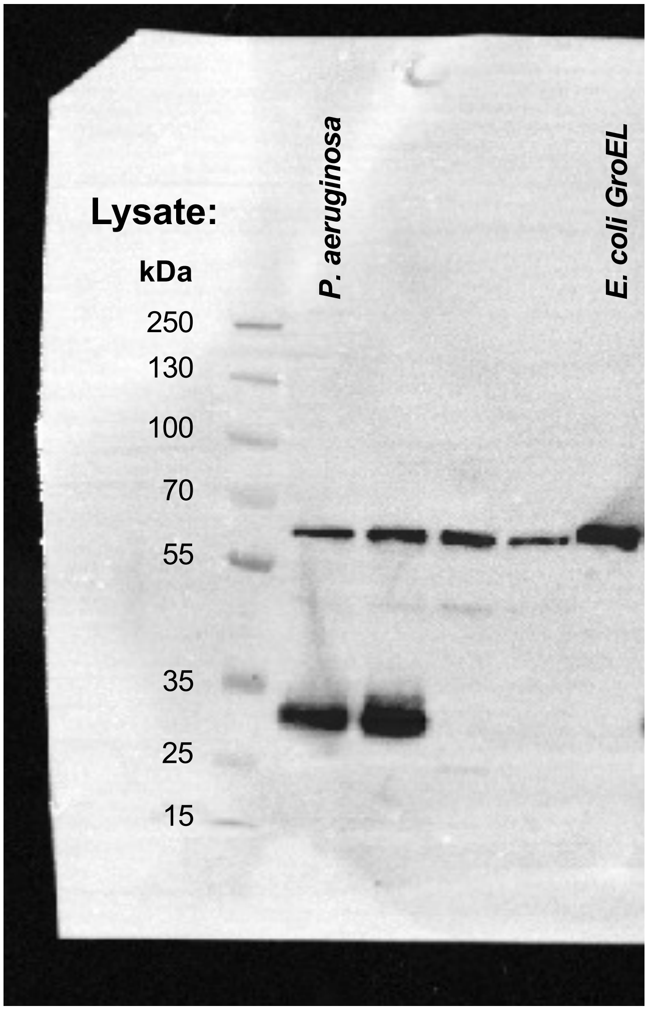

Supplemental Figure 6. GroEL is a highly conserved bacterial protein and can be bound by *Bp*-WCV induced serum antibodies. Immunoblotting of *Bp*-WCV serum to *P. aeruginosa* lysate and recombinant *E. coli* GroEL. All blots in this panel were performed in parallel.

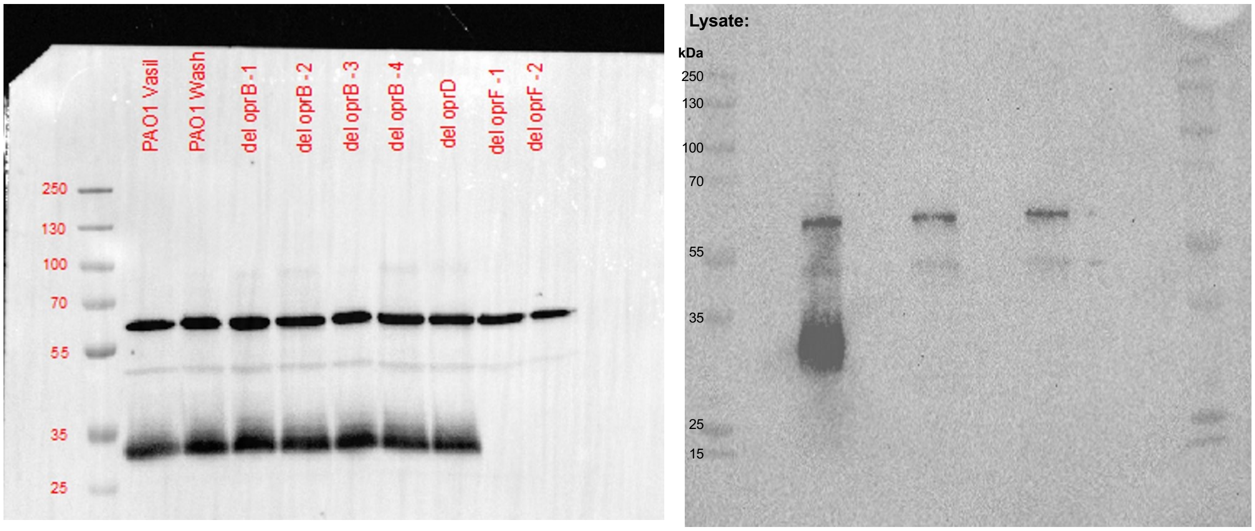

Supplemental Figure 7. Serum antibodies in *B. pertussis* whole cell immunized mice bind to *P. aeruginosa* OprF, which is homolog to *B. pertussis* OmpA. Western blot analysis of binding of cross-reactive antibodies to wild-type PAO1 and PA14 strains of *P. aeruginosa*, and to transposon mutants containing insertions in the *oprF,* as indicated in part A with an arrow. All blots in this panel were performed in parallel.

Supplementary Table 1. Bacterial strains used in this study.

Bacterial Species	Source	Notes
Escherichia coli	ATCC 25922	
Staphylococcus aureus	ATCC 6538	
Klebsiella pneumoniae	ATCC 12883	
Acinetobacter baumannii	ATCC 19606	
Enterobacter cloacae	ATCC BAA-2468	
P. aeruginosa PAO1 Vasil		
WT PAO1 Washington	Held, et al. 2012	Parental strain
PAO1 tn::*oprF*-1	Held, et al. 2012	Strain PW4134
PAO1 tn::*oprF*-2	Held, et al. 2012	Strain PW4135
WT PA14	Liberati, et al. 2006	Parental strain
PA14::oprF-1	Held, et al. 2012	Strain PA14NR: 46438
PA14::oprF-2	Held, et al. 2012	Strain PA14NR: 23102
P. aeruginosa CEC34	Burns, et al. 2001	Collected from brochioalveolar lavage, mucoid, irregular shaped large colony, >1 cm swimming, + rhamnolipid, Serotype O1
P. aeruginosa CEC38	Burns, et al. 2001	Collected from oropharyngeal swab, non-mucoid, small, round colony, >1 cm swimming, + rhamnolipid, Serotype O6
P. aeruginosa CEC45	Burns, et al. 2001	Collected from oropharyngeal swab, non-mucoid, small irregular colony, surface swarming, + rhamnolipid, Serotype O6
P. aeruginosa CEC55	Burns, et al. 2001	Collected from oropharyngeal swab, mucoid, large round colony morphology, <1 cm of swimming, + rhamnolipid, Serotype O11
P. aeruginosa CEC65	Burns, et al. 2001	Collected from oropharyngeal swab, oropharyngeal swab, large round colony, <1 cm of swimming, -/+ rhamnolipid, Serotype O6
P. aeruginosa CEC76	Burns, et al. 2001	Collected from oropharyngeal swab, mucoid, medium round colony, >1cm of swimming, + rhamnolipid, Serotype O4
P. aeruginosa CEC86	Burns, et al. 2001	Collected from bronchioalveolar lavage, small irregular colony, <1cm, + rhamnolipids, Serotype O3

Supplementary Table 2. Mass spectrometry revealed ten potential cross-reactive *P. aeruginosa* antigens bound by *Bp*-WCV induced antibodies.

Gene	Locus	Description	Coverage %	# Peptides
groEL	PA4385	60 kDa chaperonin	48	25
oprF	PA1777	Outer membrane porin F	30	7
atpA	PA5556	ATP synthase subunit alpha	11	4
hupB	PA1804	DNA-binding protein HU-beta	50	3
rplP	PA4256	50S ribosomal protein L16	23	3
atpD	PA5554	ATP synthase subunit beta	7	3
oprI	PA2853	Major outer membrane lipoprotein	25	2
oprL	PA0973	Peptidoglycan-associated lipoprotein	17	2
rplR	PA4247	50S ribosomal protein L18	21	2
arcB	PA5172	Ornithine carbamoyltransferase, catabolic	6	2
