# Supplementary material for: Bordetella pertussis whole cell immunization protects against Pseudomonas aeruginosa infections
Source: NPJ Vaccines. 2022 Nov 10;7:143. doi: 10.1038/s41541-022-00562-1 (PMC9649022; doi:10.1038/s41541-022-00562-1)
Supplement: Supplementary file 2 — REPORTING SUMMARY [file 41541_2022_562_MOESM2_ESM.pdf]

nature portfolio

Corresponding author(s): Dr Mariette Barbier

Last updated by author(s): Sep 29, 2022

Reporting Summary

Nature Portfolio wishes to improve the reproducibility of the work that we publish. This form provides structure for consistency and transparency in reporting. For further information on Nature Portfolio policies, see our Editorial Policies and the Editorial Policy Checklist.

Statistics

For all statistical analyses, confirm that the following items are present in the figure legend, table legend, main text, or Methods section.

n/a	Confirmed	
☐	☒	The exact sample size (*n*) for each experimental group/condition, given as a discrete number and unit of measurement
☐	☒	A statement on whether measurements were taken from distinct samples or whether the same sample was measured repeatedly
☐	☒	The statistical test(s) used AND whether they are one- or two-sided<br>*Only common tests should be described solely by name; describe more complex techniques in the Methods section.*
☒	☐	A description of all covariates tested
☐	☒	A description of any assumptions or corrections, such as tests of normality and adjustment for multiple comparisons
☐	☒	A full description of the statistical parameters including central tendency (e.g. means) or other basic estimates (e.g. regression coefficient) AND variation (e.g. standard deviation) or associated estimates of uncertainty (e.g. confidence intervals)
☐	☒	For null hypothesis testing, the test statistic (e.g. *F*, *t*, *r*) with confidence intervals, effect sizes, degrees of freedom and *P* value noted<br>*Give P values as exact values whenever suitable.*
☒	☐	For Bayesian analysis, information on the choice of priors and Markov chain Monte Carlo settings
☒	☐	For hierarchical and complex designs, identification of the appropriate level for tests and full reporting of outcomes
☒	☐	Estimates of effect sizes (e.g. Cohen's *d*, Pearson's *r*), indicating how they were calculated

Our web collection on statistics for biologists contains articles on many of the points above.

Software and code

Policy information about availability of computer code

Data collection	N/A
Data analysis	N/A

For manuscripts utilizing custom algorithms or software that are central to the research but not yet described in published literature, software must be made available to editors and reviewers. We strongly encourage code deposition in a community repository (e.g. GitHub). See the Nature Portfolio guidelines for submitting code & software for further information.

Data

Policy information about availability of data

All manuscripts must include a data availability statement. This statement should provide the following information, where applicable:

- Accession codes, unique identifiers, or web links for publicly available datasets
- A description of any restrictions on data availability
- For clinical datasets or third party data, please ensure that the statement adheres to our policy

Provide your data availability statement here.

Human research participants

Policy information about studies involving human research participants and Sex and Gender in Research.

Reporting on sex and gender	N/A
Population characteristics	N/A
Recruitment	N/A
Ethics oversight	N/A

Note that full information on the approval of the study protocol must also be provided in the manuscript.

Field-specific reporting

Please select the one below that is the best fit for your research. If you are not sure, read the appropriate sections before making your selection.

☒ Life sciences ☐ Behavioural & social sciences ☐ Ecological, evolutionary & environmental sciences

For a reference copy of the document with all sections, see nature.com/documents/nr-reporting-summary-flat.pdf

Life sciences study design

All studies must disclose on these points even when the disclosure is negative.

Sample size	Sample sizes were chosen based on preliminary data indicating that a large biological effect would be detectable with a minimum of 6 mice per group. In some cases, as a smaller biological effect was expected, studies were repeated to include additional animals.
Data exclusions	All data collected for each experiment performed are presented in the study.
Replication	Experiments with Category C pain with animals were performed at least twice (in some cases 3 times for some control groups) to ensure day-to-day and cohort-to-cohort consistency of the data. Experiments with Category E pain with animals were only performed once to reduce the number of animals used in these studies to the strict minimum.
Randomization	Animals were randomly selected for these studies
Blinding	These studies were not blided

Reporting for specific materials, systems and methods

We require information from authors about some types of materials, experimental systems and methods used in many studies. Here, indicate whether each material, system or method listed is relevant to your study. If you are not sure if a list item applies to your research, read the appropriate section before selecting a response.

Materials & experimental systems

n/a	Involved in the study	
☐	☒	Antibodies
☒	☐	Eukaryotic cell lines
☒	☐	Palaeontology and archaeology
☐	☒	Animals and other organisms
☒	☐	Clinical data
☒	☐	Dual use research of concern

Methods

n/a	Involved in the study	
☒	☐	ChIP-seq
☒	☐	Flow cytometry
☒	☐	MRI-based neuroimaging

Antibodies

Antibodies used	Anti-IgG secondary antibody conjugated to alkaline phosphatase (SouthernBiotech, #1030-04)<br>Anti-IgG secondary antibody conjugated to horseradish peroxidase (HRP) (Novus Biologicals, # NBP1-75130)
Validation	SouthernBiotech #1030-4 information provided on the vendor website:<br>Reacts with the heavy chains of mouse IgG1, IgG2a, IgG2b, IgG2c, and IgG3. van Ginkel FW, Wahl SM, Kearney JF, Kweon M, Fujihashi

K, Burrows PD, et al. Partial IgA-deficiency with increased Th2-type cytokines in TGF-β1 knockout mice. J Immunol. 1999;163:1951-7. (ELISA, ELISPOT)
2. Dunn LA, Upcroft JA, Fowler EV, Matthews BS, Upcroft P. Orally administered Giardia duodenalis extracts enhance an antigen-specific antibody response. Infect Immun. 2001;69:6503-10. (ELISA, IHC-FS)
3. Lagrota-Candido J, Vasconcellos R, Cavalcanti M, Bozza M, Savino W, Quirico-Santos T. Resolution of skeletal muscle inflammation in mdx dystrophic mouse is accompanied by increased immunoglobulin and interferon-γ production. Int J Exp Path. 2002;83:121-32. (ELISA, ELISPOT)
Novus Biologicals #NBP1-75130 information provided on vendor website: Based on IEP, this Goat anti-Mouse IgG (H +L) Secondary Antibody [HRP] reacts with heavy gamma chains on mouse IgG and light chains on all mouse immunoglobulins. Publications: Wong TY, Lee KS, Russ BP et al. Intranasal administration of BReC-CoV-2 COVID-19 vaccine protects K18-hACE2 mice against lethal SARS-CoV-2 challenge NPJ vaccines Mar 14 2022 [PMID: 35288576] (ELISA) and Bosnakovski D, Ener ET, Cooper MS Et al. Inactivation of the CIC-DUX4 oncogene through P300/CBP inhibition, a therapeutic approach for CIC-DUX4 sarcoma Oncogenesis Oct 12 2021 [PMID: 34642317] (ICC/IF) ICC/IF

Animals and other research organisms

Policy information about studies involving animals; ARRIVE guidelines recommended for reporting animal research, and Sex and Gender in Research

Laboratory animals	6-week old CD-1 mice females from Charles River (Strain 022), 6-weeks old males and females transgenic β-ENaC mice (kindly provided by Dr. Livraghi-Butrico, University of North Carolina) and C57B/6 littermates
Wild animals	N/A
Reporting on sex	Sex is included as a variable in studies with transgenic mice but insufficient number of mice from each sex were included to be able to perform analyses on disaggregated data.
Field-collected samples	N/A
Ethics oversight	All experiments were performed under protocols approved by the West Virginia University institutional animal use and care committee.

Note that full information on the approval of the study protocol must also be provided in the manuscript.
